# Integrated Analysis of Gene Expression, CpG Island Methylation, and Gene Copy Number in Breast Cancer Cells by Deep Sequencing

**DOI:** 10.1371/journal.pone.0017490

**Published:** 2011-02-25

**Authors:** Zhifu Sun, Yan W. Asmann, Krishna R. Kalari, Brian Bot, Jeanette E. Eckel-Passow, Tiffany R. Baker, Jennifer M. Carr, Irina Khrebtukova, Shujun Luo, Lu Zhang, Gary P. Schroth, Edith A. Perez, E. Aubrey Thompson

**Affiliations:** 1 Division of Biomedical Statistics and Informatics, Department of Health Sciences Research, Mayo Clinic College of Medicine, Rochester, Minnesota, United States of America; 2 Department of Cancer Biology, Mayo Clinic Comprehensive Cancer Center, Jacksonville, Florida, United States of America; 3 Illumina, Inc., Hayward, California, United States of America; 4 Department of Medicine, Mayo Clinic, Jacksonville, Florida, United States of America; Vanderbilt University Medical Center, United States of America

## Abstract

We used deep sequencing technology to profile the transcriptome, gene copy number, and CpG island methylation status simultaneously in eight commonly used breast cell lines to develop a model for how these genomic features are integrated in estrogen receptor positive (ER+) and negative breast cancer. Total mRNA sequence, gene copy number, and genomic CpG island methylation were carried out using the Illumina Genome Analyzer. Sequences were mapped to the human genome to obtain digitized gene expression data, DNA copy number in reference to the non-tumor cell line (MCF10A), and methylation status of 21,570 CpG islands to identify differentially expressed genes that were correlated with methylation or copy number changes. These were evaluated in a dataset from 129 primary breast tumors. Gene expression in cell lines was dominated by ER-associated genes. ER+ and ER− cell lines formed two distinct, stable clusters, and 1,873 genes were differentially expressed in the two groups. Part of chromosome 8 was deleted in all ER− cells and part of chromosome 17 amplified in all ER+ cells. These loci encoded 30 genes that were overexpressed in ER+ cells; 9 of these genes were overexpressed in ER+ tumors. We identified 149 differentially expressed genes that exhibited differential methylation of one or more CpG islands within 5 kb of the 5′ end of the gene and for which mRNA abundance was inversely correlated with CpG island methylation status. In primary tumors we identified 84 genes that appear to be robust components of the methylation signature that we identified in ER+ cell lines. Our analyses reveal a global pattern of differential CpG island methylation that contributes to the transcriptome landscape of ER+ and ER− breast cancer cells and tumors. The role of gene amplification/deletion appears to more modest, although several potentially significant genes appear to be regulated by copy number aberrations.

## Introduction

The advent of massively parallel DNA sequencers has opened new vistas on cancer genomics. Wide dynamic range and high signal to noise ratio facilitates sequence-based genomic profiling of low abundance transcripts, which cannot be reliably detected using microarrays. Deep sequence analysis of restriction endonuclease fragments from bisulfate-treated genomic DNA fragments makes it possible to quantify changes in CpG island methylation status, and low depth quantitative DNA sequence analysis provides a rapid means to identify genes that are either amplified or deleted during transformation.

However, our ability to generate detailed sequence information has significantly outstripped the power of the available analytical pipelines in many cases. A major objective of our studies has been to produce and to make publicly available a comprehensive sequence-based dataset that can be used to develop new analytical pipelines. A more daunting challenge is the development of quantitative models that describe the relationship between diverse genomic features such as mRNA abundance, epigenetic modification, and gene copy number. It is our belief that such a systems biology approach will eventually enable the incorporation of multiple genomic features into a quantitative model of the genomic landscape of individual tumors, and that such a perspective will be clinically useful for stratification of tumors for prognostic and/or predictive applications. We currently lack the ability to generate such models, but we submit that the availability of detailed sequence-based genomic datasets of the sort that we present below provides a valuable resource for the development of such analytical pipelines. To this end we have carried out deep sequence analysis of eight well-characterized human breast cancer cell lines. These data have been broadly analyzed with a view towards assessing the extent to which copy number aberration and/or differences in CpG island methylation account for differential gene expression in cohorts of cells that model clinically relevant states. Specifically, we have focused on comparison of a panel of breast cancer cell lines that either express or do not express estrogen receptor-α (the product of the *ESR1* gene, hereinafter called ER).

Several studies using cDNA/oligonucleotide microarray or SAGE (serial analysis of gene expression) have shown that ER+ and ER− breast cancers have very different gene expression profiles that can be used for molecular diagnosis and outcome prediction [1]–[4]. These findings suggest that a subset of genes co-expressed with ER could play an important role in establishment and maintenance of the transformed state and in determining the hormone-responsive breast cancer phenotype [5]. However, the underlying mechanisms that account for differential regulation and function of these genes are largely unknown. In this study we applied next generation cDNA sequencing technology (mRNA-seq) to quantify mRNA abundance and identify genes that are differentially expressed in a panel of well-characterized ER+ and ER- cell lines at a depth of analysis that has not yet been achieved by conventional microarray analyses. Low depth DNA sequence analysis (DNA-seq) was used to quantify gene copy number to a depth of about 1 sequence tag every 300 bp, with a view towards determining if there are common patterns of gene amplification or deletion that underlie aspects of the genomic profiles that are associated with the ER+ or ER− phenotypes. Finally, bisulfite-treated DNA fragments (Methyl-seq) were sequenced to quantify changes in CpG island methylation and to determine if there are systematic patterns of methylation that may contribute to differential gene expression in ER+ versus ER− cells. These analyses were carried out simultaneously for 7 commonly used breast cancer cell lines (MCF7, T47D, BT474, ZR75-1, BT20, MDA-MB-231, MDA-MB-468) and 1 non-tumor breast cell line (MCF10A). This dataset represents one of the most comprehensive genomic portraits of a collection of tumor cell lines reported to date; and, from an analytical perspective the dataset has considerable utility for developing analytical pipelines to mine sequence-based genomic data as well as to evaluate the relative contributions of promoter methylation and gene copy number aberrations in defining patterns of gene expression. Our initial analysis has identified a cohort of genes that are differentially expressed in ER+ and ER− cell lines, associated with changes in gene copy number or CpG island methylation status in such cells, and differentially expressed in ER+ and ER− primary human breast tumors. Several of these genes have been implicated in hormone responsiveness and disease progression.

## Results

### mRNA expression patterns are associated with ER status

**Figure 1:**
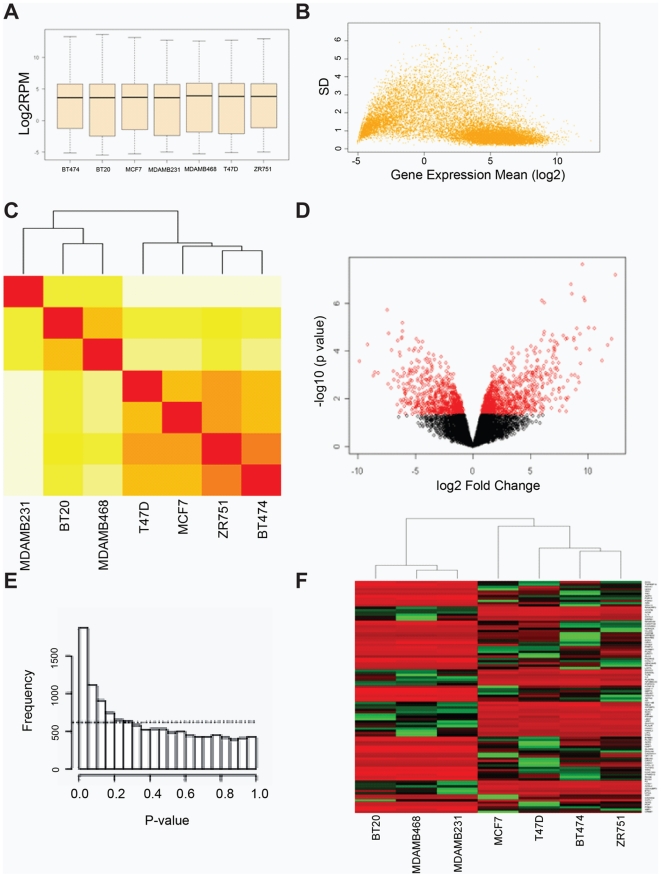
Total mRNA sequence analysis identifies a cohort of genes that are differentially expressed in ER+ and ER− cell lines. **Panel A**: A box plot of total read normalized (RPM) log2 transformed data for 7 breast cancer cell lines. **Panel B**: RPM mean versus standard deviation (SD) of 7 cell lines showing variation is much higher in low abundance transcripts. Log2 = 0 corresponds to ∼1 RPM or about 50 raw counts. **Panel C**: An unsupervised clustering using all genes for 7 cell lines. The graded colors from red, orange, to yellow represent correlation from high to low among samples. ER+ and ER− cell lines are in two different clusters. **Panel D**: A volcano plot for differentially expressed genes identified using LIMMA statistical model. The red circles indicate genes significant at p<0.05 and fold change >1.5. **Panel E**: p-value distribution of all genes in the analysis indicates that p-values are not uniformly distributed, as would be predicted if the distribution of p-values were random. Random frequency distribution was approximated by assuming that if the distribution were random, the p-values for individual genes would be uniformly (equally) distributed across the different bins of p-values. From this assumption, we estimated the number of genes that would distribute to each bin by dividing the total number of genes by 20 bins. This calculation estimates a random frequency of ∼624 genes in each p-value bin simply by chance, as indicated by the dashed line. **Panel F**: A heatmap showing the strict assortment of ER+ and ER− tumors based on the top 100 differentially expressed genes identified using the LIMMA model. Gene expression was standardized by the mean among the samples, red indicating higher expression and green for lower expression.

We carried out 50 nt paired end mRNA-seq analysis on 8 cell lines. Seven cancer cell lines were used in this comparison: 4 ER+ and 3 ER−. The non-tumorigenic cell line MCF10A was excluded in our initial comparison of mRNA abundance in tumor cell lines. Initially, we identified and excluded 710 genes that had 0 mapped reads in all samples, when mapped to the 18,517 annotated genes in the RefSeq RNA database (release 30, 2008). The reads/million (RPM) normalized, log2 transformed data for the remaining 17,807 exhibited very similar distribution from sample to sample when displayed as a box plot (Figure 1A). As expected, genes that were expressed at low levels were more variable among cell lines, as shown in the mean versus standard deviation plot (Figure 1B). Unsupervised clustering using all genes (Figure 1C) demonstrated that ER+ and ER− cell lines formed two distinct clusters consistent with published microarray data from tumor samples [1], [4]. Similar results were obtained when unsupervised clustering was carried out with a subset of 4450 genes with standard deviation above 75 percentile (data not shown). Because of the small number of samples, we were concerned that the hierarchical relationship shown in Figure 1C might be random. To evaluate this possibility, we carried out 100 iterations in which we perturbed by adding artificial noise to the dataset as described by McShane and colleagues [6]. The noise was estimated from the dataset by calculating the variance of each gene among the cell lines and then using the median of these variances to define the variance of the noise, which were then randomly selected and added to the original data for re-clustering 100 times. At the point of two clusters that separated ER+ and ER− cell lines, we obtained an R (Robustness) index of 1 and a D (Discrepancy) index of 0, suggesting the strong reliability of the clusters.

We applied two additional filters to eliminate variable genes that were expressed at low abundance (Figure 1B). Initially, we filtered the 17,807 genes to eliminate those with average raw counts less than 50 (mean RPM∼≤1, corresponding to log2 mean expression = 0 in Figure 1B) in both ER+ and ER− groups. We also required that every gene, in addition to having >50 average raw counts in one group of samples, must also have at least 5 raw counts in every sample in that same group. These filters reduced the dataset to 12,487 genes, of which 1,873 were differentially expressed in ER+ and ER− cancer cell lines, as defined by p<0.05 (FDR q<0.2) from moderated (LIMMA) t-statistics and fold change greater than 1.5. A ‘volcano plot’ illustrating differential expression (red symbols) as a function of fold change and p-value is shown in Figure 1D. Figure 1E illustrates the frequency distribution of p-values for this dataset, which manifests a peak frequency in the range of 0≤p≤0.05. The reference line in Figure 1E illustrates the p-value distribution that would be expected if differential expression reflects random chance. The identities of these 1,873 differentially expressed genes as well as the LIMMA statistics that support their identification are provided in Table S1. The heatmap shown in Figure 1F contains the top 100 differentially expressed genes (defined by p-value) and illustrates the consistency with which this subset of genes were differentially expressed in the ER+ and ER− cell lines.

### Validation of mRNA-seq data

**Figure 2:**
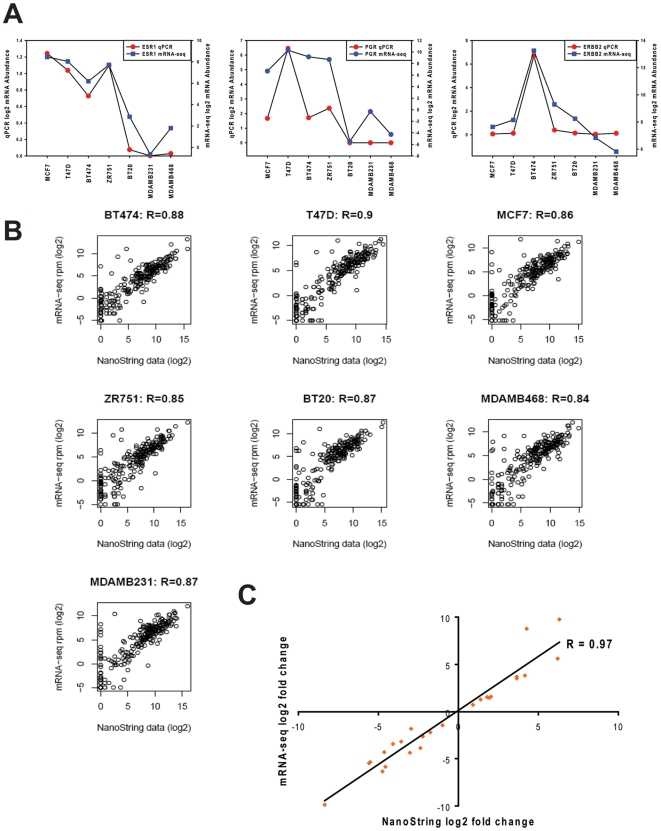
The mRNA-seq data validate in comparison to qPCR and NanoString data. **Panel A**: A comparison of the abundance of three transcripts (ESR1, PGR, and ERBB2) measured by mRNA-seq (blue symbols) or qPCR (red symbols). **Panel B**: A correlation plot between mRNA-seq and NanoString for 236 cancer reference genes. Log2 RPM data for mRNA-seq and log2 NanoString data were used. R was used to calculate the Pearson correlation coefficient. **Panel C**: log2 fold change correlation between mRNA-seq and NanoString for 25 differentially expressed genes detected by NanoString.

Initially, we compared the abundance of the three sentinel markers of our cell lines (ER/ESR1, PR/PGR, and HER2/ERBB2) using qPCR data that we had originally generated to confirm the receptor status of the cell lines. As shown in Figure 2A, there was generally good correlation between the qPCR and mRNA-seq data for these three transcripts. We then used the NanoString nCounter and the Cancer Reference codeset to extend our validation to 236 cancer-related genes. The RPM-normalized mRNA-seq total counts for each of these genes are shown in comparison to the nCounter counts in Figure 2B. Very high correlations were observed between mRNA-seq and NanoString data for each of the 7 cancer cell lines (Pearson correlation coefficients ranging from 0.84 to 0.9).

The NanoString codeset included 25 genes that were scored as differentially expressed (p<0.05) in the mRNA-seq data. The expression data for these genes is plotted as log2 fold change (ER+ versus ER−) in Figure 2C, which shows a very high degree of correlation (R = 0.97) between the NanoString and mRNA-seq dataset. The data indicate the quantitative precision of the sequence-based approach for identification of differentially expressed transcripts.

### Differentially expressed genes are involved in ER-associated pathways

We conducted a pathway analysis for 451 genes with q-value less than 0.1 (corresponding to p-value less than 0.005) and log2 fold change>±1.3. Twenty seven canonical pathways and 25 biological process networks were significantly enriched at p-value less than 0.05, as listed in Table S2. We noted that several significantly enriched pathways/networks are linked to ER function or expression, consistent with the receptor status of the cell lines.

### Global methylation patterns correlate with ER status

**Figure 3:**
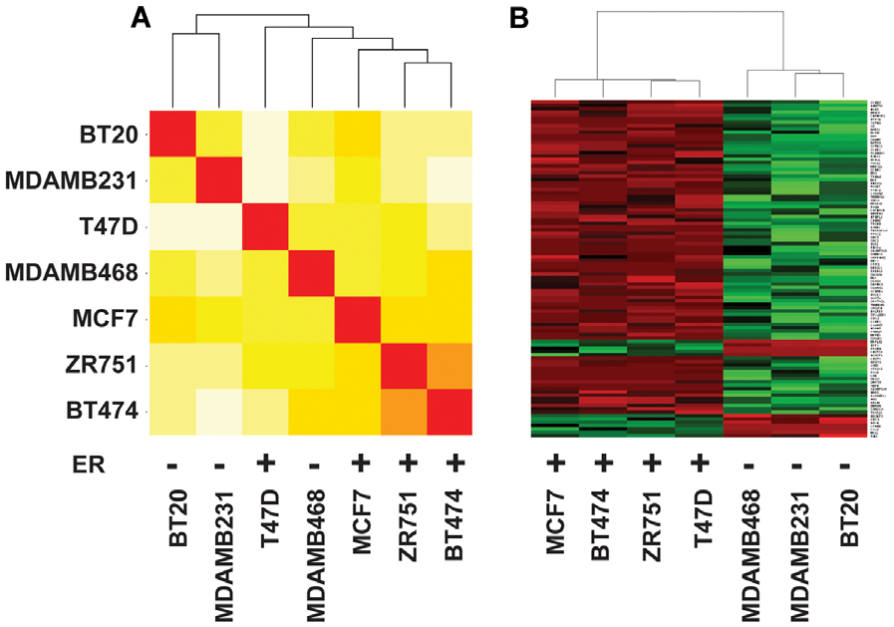
ER+ and ER− cell lines exhibit differential CpG island methylation. **Panel A**: Unsupervised clustering of cell line data using 21,570 CpG islands, filtered for CMP methylation coverage as described in Materials and Methods. The graded colors from red, orange, yellow, to white represent correlation from high to low among samples. **Panel B**: A heatmap of the top 100 differentially methylated CpG islands identified using the LIMMA model. The methylation data for each CpG island were standardized by mean among the samples where red represents hypermethylation and green hypomethylation. Genes associated with these CpG islands are indicated on the right of the figure.

The human genome contains 25,328 annotated CpG islands with overlapping *MspI* restriction sites. For purposes of analysis, we required at least 10× coverage of each dCMP residue within every CpG island, and the average of all dCMP residues within each island was calculated as described in the Materials and Methods. We analyzed 21,570 CpG islands that met these criteria in all seven breast cancer cell lines, which represents 85% of the total *MspI*-bounded CpG islands in the genome. We conducted unsupervised clustering using the methylation data of all 21,570 CpG islands. As shown in Figure 3A, three ER+ cell lines BT474, ZR751, and MCF7, were in the neighboring nodes of the cluster, and two ER- cell lines BT20 and MDAMB231 clustered. However, T47D (ER+) and MDAMB468 (ER-) did not cluster with ER+ and ER− samples, respectively. The data suggest that global genomic methylation status varies in a systematic manner between ER+ and ER− cell lines; however, the association between CpG island methylation and ER status is not as robust as that observed for mRNA abundance (Figure 1C).

Among the 12,487 genes that were included in the mRNA expression analysis, 9,968 had known CpG islands that mapped within 5 kb of the genomic coordinates of the 5′ end of the longest known transcript. About 4% of these islands (444) were differentially methylated (p<0.05) in ER+ versus ER− cells. The top 100 differentially methylated CpG islands, determined by rank order p-value, were visualized in a heatmap (Figure 3B), which illustrates a very robust methylation signature for these CpG islands in the ER+ and ER− cell lines. All significantly methylated CpG islands and their associated mRNA expression data are provided in Table S3.

**Figure 4:**
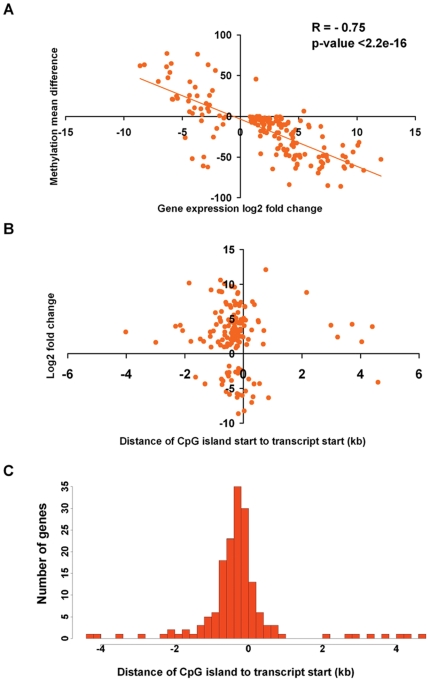
There is an inverse correlation between methylation status of promoter-proximal CpG islands and mRNA abundance. **Panel A**: A scatter plot and trend line between fold change of gene expression and mean difference of methylation between ER+ and ER− cell lines. The Pearson correlation coefficient R is −0.75 [95%CI: −0.81, −0.68] with p-value<2.2e-16. **Panel B**: The distance from the start of each of the CpG islands that exhibited inverse correlation with mRNA abundance, illustrated in Figure A, to the start of the corresponding gene plotted against log2 gene expression fold change between ER+ and ER− cell lines. **Panel C**: A histogram representing the distribution of differentially methylated CpG islands in 149 differentially expressed genes.

Of the 444 CpG islands that were differentially methylated in ER+ and ER− cell lines, 164 islands were located within 5 kb of the promoters of 162 differentially expressed genes. The relationship between the mean methylation difference and the log2 fold change for those 162 genes is shown in Figure 4A (Pearson correlation coefficient = −0.75 with 95% confidence interval: −0.81 to −0.68 and regression p-value<2.2e-16). A strong negative correlation was observed between methylation status and expression for most of these genes, although there were a number of outliers (13 genes) in which methylation status appeared to be unrelated to expression. We observed a strong inverse correlation between methylation status of 151 CpG islands and expression of 149 associated genes.

These differentially expressed genes also exhibited an inverse relationship between the magnitude of differential gene expression (log2 fold change) and distance of a CpG island to the cognate transcript start site (Figure 4B) indicating that proximity to the promoter was a major factor in determining the extent to which gene silencing was linked to promoter methylation. Analysis of the distribution of CpG islands with respect to the 5′ end of the differentially expressed transcripts indicates that the median distance from the differentially methylated CpG islands to the transcriptional start site is around 300 bp (Figure 4C), consistent with a role in affecting promoter activity. These 149 genes are listed in Table 1, with detailed expression data in Table S4. The strong inverse correlation between methylation status of these promoter-proximal CpG islands and mRNA abundance of the cognate transcripts is consistent with the conclusion that a subset of those genes that define the ER+ and ER− gene expression profiles are likely to be regulated by tumor subtype specific changes in the global pattern of CpG island methylation.

**Table 1:** 149 genes differentially expressed and inversely correlated with CpG island methylation.

Genes overexpressed in ER+ cell lines and hypermethylated in ER− cell lines (117)				Genes overexpressed in ER− tumors and hypermethylated in ER+ cell lines (32)
***ACOT4***	*CUX2*	*MPPED2*	*SEPT5*	***ACN9***
***ADAMTS13***	***CXCL12***	*MPV17L*	***SIDT2***	***ADORA2B***
*ADAMTS19*	***CXXC5***	*MSI1*	***SLC16A6***	*AKR1B1*
***ADCY1***	***DNAJA4***	***MYRIP***	*SLC16A9*	***ALDH1A3***
***AMPH***	*DSCAML1*	*NAAA*	***SLC1A2***	*ANKH*
*AMZ2*	*ENTPD2*	***NKD2***	*SLC29A4*	*CAV2*
***AR***	*FGFR4*	***NOVA1***	*SLITRK4*	***CHST11***
*ASCL2*	***FKBP4***	***NPEPL1***	*SPATA2L*	***CPNE8***
***ASTN2***	*FSCN2*	***NPNT***	***SPATA7***	***EGFR***
***ATP2A3***	***GATA3***	*NPTXR*	*SPNS1*	***EPHB2***
***ATP8B2***	***GFRA1***	*P2RX2*	*SRMS*	***FMNL2***
***BTG2***	***GHR***	***PALM***	***ST6GALNAC2***	*FOSL1*
*C16orf14*	***GJD3***	***PATZ1***	***STK32B***	*GPX1*
***C17orf28***	***GPR160***	*PAX9*	***STOM***	*HS3ST1*
*C20orf134*	*GRIK3*	*PCP4L1*	*SYCP2*	***IGF2BP2***
***C3orf57***	***HS6ST3***	*PDZRN3*	*TMEM47*	***IGF2BP3***
*C6orf154*	*ID2*	***PGR***	*TRPV4*	*KIFC3*
***C6orf97***	***IFT140***	*PLCB1*	***VPS37D***	***LYN***
*CA8*	***IGFBP2***	***PRKCZ***	*ZNF396*	***MALL***
***CACNA1H***	***IGSF9B***	***PRKG1***	***ZNF512B***	***MSN***
***CACNA2D2***	*IL17RB*	*PRUNE2*	***ZNF703***	*NEXN*
*CADM1*	***JAM2***	*PSTPIP2*		*NPAS2*
***CAMK2B***	*KCNH1*	***PTGER3***		***PLAC8***
*CASKIN1*	***KCNK6***	*RAPGEFL1*		*PPARG*
***CELSR1***	***KCNMA1***	***RHBG***		*RIN3*
*CGREF1*	***KIF12***	***RHOT2***		*SEMA7A*
***CHDH***	***KIFC2***	*RICH2*		*SLC19A3*
*CHST1*	***KLHDC9***	*RND2*		*TEC*
***CLUAP1***	***LMX1B***	***RNF40***		***TXNRD1***
*CNTNAP2*	*LRRC26*	*SAMD11*		***UPP1***
***CPLX1***	***MAPK8IP2***	*SDC2*		***VLDLR***
***CRIP2***	***MMP17***	***SEMA6A***		*ZNF502*

Genes in bold face were also differentially expressed between 76 ER+ and 53 ER− breast tumors and were consistent with cell line expression and methylation data. Among these, 67 were overexpressed in ER+ tumors and 17 were overexpressed in ER− tumors. Detailed gene expression and methylation data for the 149 genes (153 CpG islands) in the cell lines can be found in Table S4.

### Differential gene amplification and deletion is related to ER status

**Figure 5:**
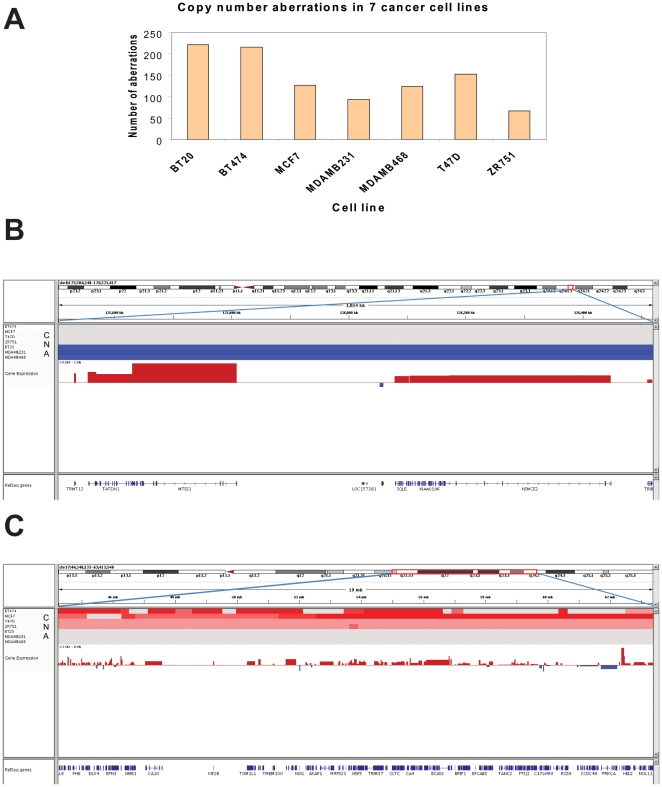
Gene copy number aberrations are associated with differential gene expression. **Panel A**: A histogram of the number of statistically significant CNAs identified in each cell line. **Panel B**: IGV (Integrative Genomics Viewer) view of copy number aberrations for the region of chromosome 8 that is deleted in all three ER− cell lines. **Panel C**: Genomic view of copy number aberrations for the regions of chromosome 17 with amplification in all four ER+ cell lines. The symbols and abbreviations in Panels B and C are as follows: CNA - the copy number aberration track for each individual cell line; red represents amplification, blue deletion, and gray no change. Gene expression – the differential gene expression track; red represents overexpression in ER+ cells, blue represents overexpression in ER− cells, shown as log2 fold change.

DNA-seq analysis was carried out as described in Materials and Methods to a theoretical coverage of one tag distributed approximately every 300 across the genome. Copy number aberrations were identified by comparing the number of sequence tags that aligned to the genome in each tumor cell line to non-tumorigenic MCF10A cells. Every cell line exhibited 100–200 statistically significant copy number differences, compared to MCF10A (Figure 5A). We identified 1,003 genomic regions that exhibited statistically significant copy number changes (Table S5) in one or more of the cancer cell lines. We focused our analysis on 799 copy number aberrations that were found in at least two of the samples; 479 (out of 1,873) differentially expressed genes were mapped to these regions, suggesting that differential expression, in some cases, might result from gene copy number differences. However, comparison of the log2 fold change of the mRNA between ER+ vs. ER− sample groups that corresponds to these 479 differentially expressed genes and the log2 ratio copy number difference of the corresponding genomic coordinated in ER+ and ER− sample groups showed only moderate positive correlation (correlation coefficient = 0.28). This rather weak correlation probably reflects the fact that most copy number aberrations appear to be cell specific and not strictly associated with the ER status.

We did identify regions on chromosome 8 and 17 that were consistently amplified or deleted in either ER+ or ER− cell lines. These included a region from 125,504,248 to 126,521,417 on chromosome 8 wherein all three ER− cell lines exhibited statistically significant copy number loss with no chromosome copy number change in 4 ER+ cell lines. These data are summarized in Figure 5B, which compares gene copy number in ER+ and ER− cell lines (blue = deleted in ER−, gray = no change in ER+). Eight genes mapped to this locus exhibited significantly higher expression in ER+ cell lines (red = overexpressed in ER+).

All ER+ cell lines exhibited gene amplification in chromosome 17 from 44,246,133 to 63,413,540 with no change in copy number in any of the ER− cell lines (Figure 5C, red = amplified in ER+, gray = no change in ER−). This locus contains 22 genes that were more abundant in ER+ cell lines (Figure 5C, red = overexpression in ER+ relative to ER−). A few genes within this locus appeared to be overexpressed in ER− (as indicated by blue bars), however none of these achieved statistical significance at the level of p<0.05. Overall, some 30 genes appear to be consistently overexpressed as a result of chromosome 8 deletion in ER− cells or chromosome 17 amplification in ER+ cells. These genes are listed in Table 2, with detailed expression and amplification data in Table S6.

**Table 2:** 30 differentially expressed genes in the consistent CAN.

		Gene expression		Copy number (log2 ratio relative to MCF10A)						
Chr	Genes	log2 FC*	p value	BT474	MCF7	T47D	ZR751	BT20	MDAMB231	MDAMB468
8	*TRMT12*	1.732	0.012	0	0	0	0	−1.216	−2.166	−1.326
**8**	***RNF139*** **	1.997	0.003	0	0	0	0	−1.216	−2.166	−1.326
8	*TATDN1*	1.620	0.031	0	0	0	0	−1.216	−2.166	−1.326
8	*NDUFB9*	1.608	0.004	0	0	0	0	−1.216	−2.166	−1.326
8	*MTSS1*	3.582	0.009	0	0	0	0	−1.216	−2.166	−1.326
**8**	***KIAA0196***	1.317	0.010	0	0	0	0	−1.216	−2.166	−1.326
8	*NSMCE2*	1.368	0.027	0	0	0	0	−1.216	−2.166	−1.326
17	*ATP5G1*	1.187	0.046	2.032	1.062	0.650	0.697	0	0	0
17	*UBE2Z*	1.425	0.044	2.032	1.062	0.650	0.697	0	0	0
17	*B4GALNT2*	4.600	0.010	2.032	1.062	0.650	0.697	0	0	0
17	*PHOSPHO1*	3.221	0.003	2.032	1.062	0.650	0.697	0	0	0
**17**	***NXPH3***	3.520	0.004	2.032	1.062	0.650	0.697	0	0	0
**17**	***SPOP***	1.357	0.049	2.032	1.062	0.650	0.697	0	0	0
17	*SLC35B1*	1.162	0.019	2.032	1.062	0.650	0.697	0	0	0
**17**	***TOM1L1***	1.902	0.026	2.616	0.912	0.650	0.697	0	0	0
17	*HLF*	3.270	0.021	2.616	0.912	0.650	0.697	0	0	0
17	*MKS1*	1.884	0.004	1.204	1.551	0.650	0.937	0	0	0
17	*SUPT4H1*	1.285	0.025	1.204	1.551	0.650	0.937	0	0	0
17	*MTMR4*	1.037	0.040	1.204	1.551	0.650	0.702	0	0	0
17	*RAD51C*	1.668	0.017	2.287	3.534	0.650	0.702	0	0	0
17	*TRIM37*	2.080	0.048	2.287	3.534	0.650	0.702	0	0	0
17	*C17orf71*	2.607	0.020	2.717	1.984	0.650	0.702	0	0	0
17	*DHX40*	1.586	0.021	2.717	1.984	0.650	0.702	0	0	0
**17**	***CLTC***	1.458	0.025	2.717	1.984	0.650	0.702	0	0	0
17	*TUBD1*	1.945	0.033	2.717	3.914	0.650	0.702	0	0	0
17	*RPS6KB1*	2.202	0.037	2.717	3.914	0.650	0.702	0	0	0
**17**	***APPBP2***	2.577	0.019	2.372	3.914	0.650	0.702	0	0	0
17	*GNA13*	1.110	0.041	1.633	1.489	0.650	0.702	0	0	0
17	*PITPNC1*	1.569	0.020	0.698	1.489	0.650	0.702	0	0	0
**17**	***BPTF***	1.439	0.014	0.698	1.489	0.650	0.702	0	0	0

*mRNA-seq log2 fold change between 4 ER+ and 3 ER− cell lines.

**Genes in bold face are significantly up-regulated (p≤0.05) in ER+ tumors in the set of 129 breast tumor samples.

### The methylation/expression signature defined in cell lines is significantly enriched in a cohort of breast cancer samples

A recent genomic methylation analysis of 12 ER+ and 12 ER− tumors [7] identified 5 loci that are consistently hypermethylated in one or the other tumor type (*MANEAL*, *PER1*, *NAV1*, *FAM124B*, and *ST6GALNAC1*). Among these only *MANEAL*, *NAV1*, and *PER1* had CpG islands within 5 kb of the 5′ end of the gene, and only the CpG island associated with the *NAV1* promoter was differentially methylated (p = 0.019, difference in percent methylation = 52.3%) in our cell lines. *NAV1* was also differentially expressed (log2 fold change = 4.1); however, the increased NAV1 mRNA was observed in cells in which this CpG island was hypermethylated, so this gene is unlikely to be regulated by methylation of this particular site.

The published global methylation data are consistent with our results on global CpG island methylation in cell lines (Figure 3A); the differences in global methylation patterns are not as robust as the differential expression data, and it is generally difficult to predict gene expression patterns in ER+/ER− tumor samples based on overall genomic methylation patterns. However, a much more robust methylation/expression signature emerges when one combines differential expression and differential CpG island methylation data. Our analyses identified a cohort of 149 methylated genes that contribute to the genomic profiles of ER+ and ER− breast cancer cell lines. We organized 148 of these methylation signature genes into a geneset. [One methylation signature gene, LRCC26, was not represented on the Affymetrix and was not included in our analysis.] This geneset was then used to carry out geneset enrichment analysis (GSEA) of an open source dataset derived from Affymetrix microarray analysis of 76 ER+ and 53 ER− primary breast tumors [8] (GEO accession number: GSE5460). The 148 gene methylation geneset was significantly enriched between ER+ and ER− tumors, as evidenced by a normalized enrichment score = 1.95, p<0.001, with FDR q = 0.063. By way of comparison, a geneset of 30 known ER target genes [9] exhibited a normalized enrichment score = 2.01, p = 0.002, FDR q = 0.037 in this dataset from ER+/ER− tumors.

**Figure 6:**
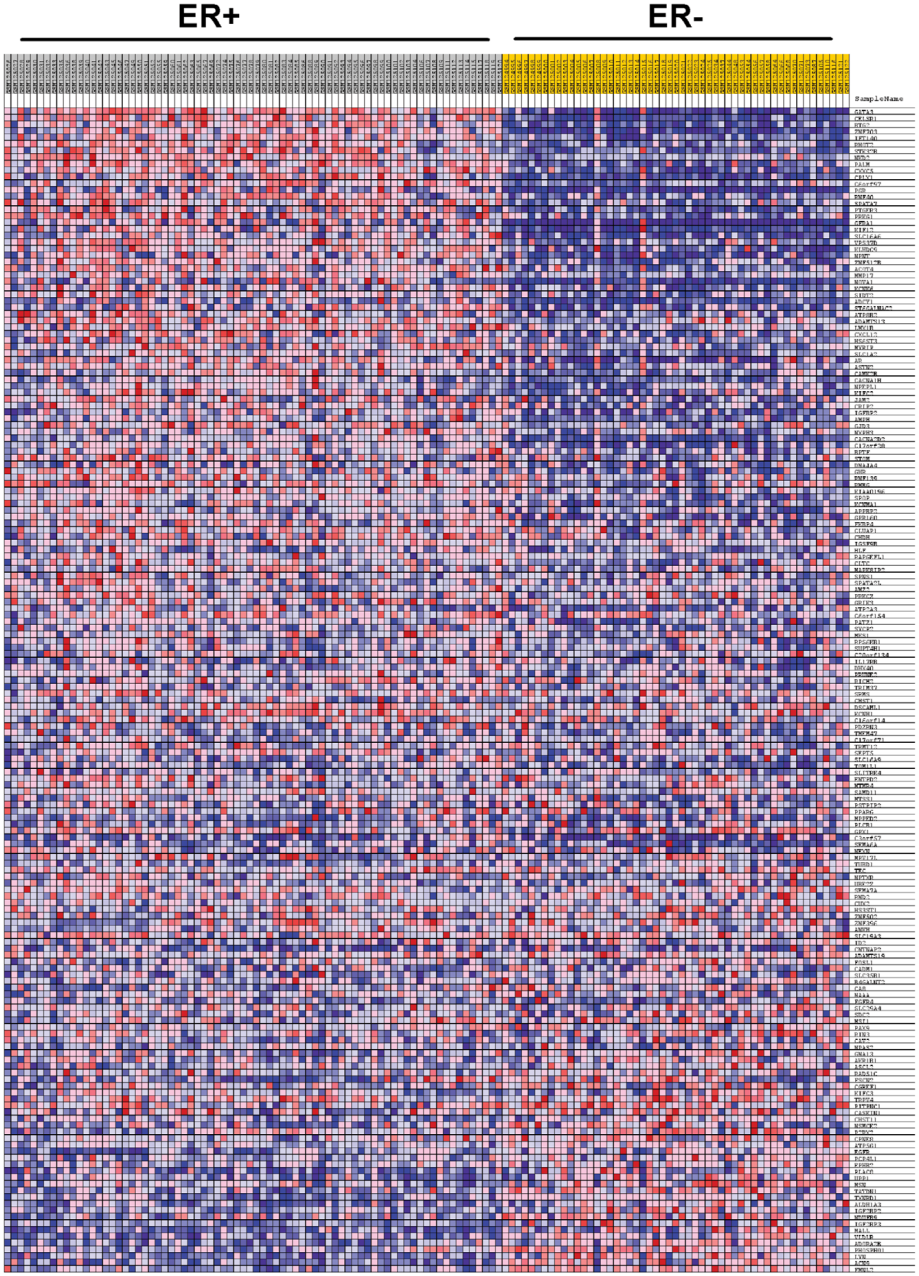
Expression of focus genes from cellular analyses in primary human breast cancer. The heatmap was generated from GSEA analysis in which microarray data from 76 ER+ and 53 ER− tumors were interrogated with a geneset consisting of 149 genes that were differentially expressed and inversely methylated plus 30 genes that were overexpressed in ER+ cells and amplified in ER+ cells or deleted in ER− cells.

A heatmap representing the expression of the 148 methylation signature genes plus 30 CNA candidate genes (identified as described above) in the tumors samples reveals the extent to which our focus genes are differentially expressed in ER+/ER− primary tumors (Figure 6). We identified 67 genes that were i) overexpressed in ER+ cell lines, ii) hypermethylated in ER− cell lines, and iii) overexpressed in primary ER+ tumors. In addition, 17 genes were i) overexpressed in ER− cell lines, ii) hypermethylated in ER+ cell lines, and iii) overexpressed in ER− primary tumors (Table 1). We conclude that combining CpG island methylation and expression data is a powerful way to identify a robust methylation/expression signature that defines at least part of the transcriptome landscape of ER+ and ER− breast cancer cells and tumors. Many of these genes are potentially important for establishment and/or maintenance of the tumor phenotype.

**Figure 7:**
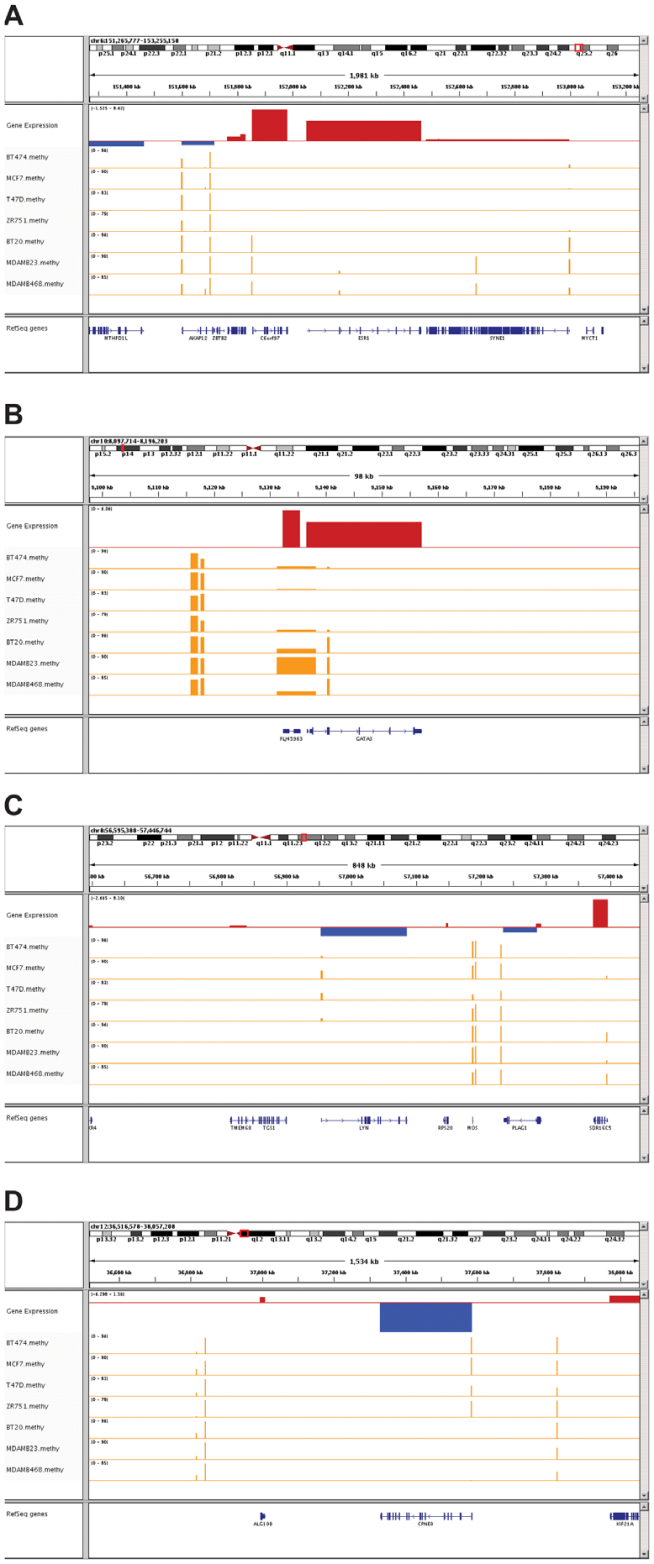
Representative examples of methylation status and mRNA abundance for genes that are differentially methylated in cell lines and differentially expressed in both cell lines and tumors. **Panel A**: *C6orf97* (with *SYNE1* and *ESR1*); **Panel B**: *GATA3*; **Panel C**: *LYN*; and **Panel D**: *CPNE8*. On each figure, gene expression track represents the log2 fold change of gene expression between ER+ and ER− cell lines, red for up-regulation and blue for down-regulation in ER+ cell lines. Below that track is the methylation data for each cell line, which shows the average percent of methylated CpGs (dynamic range 0–100%) in the CpG Islands that were interrogated in this analysis.

Figure 7 shows examples of two genes (*C6orf97 and GATA3*) that are hypermethylated in ER− cells and overexpressed in ER+ cells and tumors. Among this cohort, the greatest difference in methylation status was exhibited by *C6orf97* (Figure 7A). All three ER− cell lines exhibited almost complete methylation (mean difference %CpG methylation ER+ minus mean difference %CpG methylation ER− = −86%) of a single CpG island located adjacent to the *C6orf97* promoter (p = 6.1E-05); and *C6orf97* mRNA was very significantly overexpressed in ER+ tumors (p = 2.9E-12). The function of *C6orf97* is unknown. However, two recent large scale genome-wide association studies identified single nuclear polymorphisms (SNPs) in or near this locus which are associated with increased breast cancer risk [10], [11]. *SYNE1*, located near *C6orf97* on chromosome 6, also exhibited hypermethylation in ER− cell lines, but this gene was not differentially expressed in the tumors analyzed in this study. *C6orf97* also resides adjacent to *ESR1*, which did not exhibit a statistically significant difference in methylation status in our cell lines (Figure 7A).

High level expression of *GATA3* in ER+ tumors has been reported in a series of tissue-based studies [1], [3], [12]; and *GATA3* overexpression is associated with favorable clinical outcomes, including response to endocrine therapy [13], [14]. GATA3 mRNA is significantly overexpressed in ER+ tumors (p = 2.10E-30). As shown in figure 7B, there is a cluster of CpG islands within the *GATA3* gene that was highly methylated in ER− cells, with little or no evidence of methylation in ER+ cells (p = 2.54E-06). An uncharacterized locus (*FLJ45984*) resides immediately upstream of *GATA3* (Figure 7B), and this gene also showed evidence of differential methylation and differential expression in cell lines. Some of the CpG islands in this vicinity appear to overlap the *GATA3* promoter and may be of regulatory significance to that promoter.

We identified 17 genes that exhibited hypermethylation in ER+ cell lines and were overexpressed in ER− cell lines and tumors. CpG island methylation status of two such genes (*LYN and CPNE8*) is shown in Figure 7. *LYN* (Figure 7C) exhibited preferential methylation of a promoter-proximal GpG island in ER+ cell lines (p = 0.036), and *LYN* mRNA was consistently overexpressed in ER- tumors (p = 1.05E-09). High level expression of *LYN*, a member of the SRC family of non-receptor tyrosine kinases, has been associated with epithelial/mesenchymal transition and invasion by breast cancer cells and with poor survival of breast cancer patients [15]. Our data suggest that suppression of *LYN* expression in ER+ tumors may be linked to an epigenetic program that limits metastasis and favors better clinical outcome.

Figure 7D shows differential methylation of *CPNE8* (p = 0.007) in ER− cells. Among the genes that exhibited differential methylation, *CPNE8* exhibited the greatest difference in hypermethylation status in ER+ cell lines. A single promoter-proximal CpG island was highly methylated in the ER+ cell lines, with no evidence of methylation in the ER− cells (mean difference 77%). *CPNE8* is a member of the copine family of calcium-dependent phospholipid-binding proteins. The function of *CPNE8* is unknown, although other copine family members have been implicated in HER2 signaling and invasion in breast cancer [16]. *CPNE8* was differentially expressed in ER− tumors (p = 4.95E-04); and, given that our data suggest that CPNE8 is a very strong component of the methylation signature of ER− cells, the function of this gene warrants additional consideration.

### Combination of copy number aberration and expression data defines a CNA/expression signature that is partially reflected in ER+ and ER− tumors

A recent array comparative genomic hybridization study of 103 breast tumors identified a number of loci that exhibit copy number aberration at a significant frequency in tumor samples [17], and a significant number of these mapped to chromosome 8 or 17. Overall, 112 genes exhibited copy number aberration in 50 or more tumors. We carried out a meta-analysis to determine if the products of these 112 genes were differentially enriched in ER+ or ER− tumor samples. GSEA analysis, using the tumor dataset described above, revealed no significant enrichment (normalized enrichment score = −0.81, NOM p-value = 0.625, FDR q-value = 0.77). Overall, the array comparative genomic hybridization data and our sequence-based analysis of copy number aberration suggest that CNA varies widely from tumor to tumor and from cell line to cell line such that only a small subset of genes that consistently define the ER+ and ER− states are likely to be regulated by amplification or deletion.

In contrast, we were able to combine our CNA and expression data to identify 30 genes that were overexpressed in ER+ cell lines as a result of chromosome 17 amplification in ER+ cells or chromosome 8 deletion in ER− cells. Nine of these genes were overexpressed in ER+ tumors (at p≤0.05), as one would anticipate if these genes were deleted in ER− tumors or amplified in ER+ tumors. Two of these genes are on chromosome 8q24.13 (*RNF139* at 125,487,008 and *KIAA0196* at 126,036,502 ). *RNF4* , a nuclear receptor coregulator with ubiquitin ligase activity [18] [19], is known to be translocated in renal tumors [20] and has been implicated in ovarian cancer [21]. *NXPH3*, *SPOP*, *TOM1L1*, *HLF*, *CLTC*, *APPBP2*, and *BPTF* are located within 20 Mbp of each other on chromosome 17 between 46,970,127 and 65,821,640 (start to pter). *NXPH3* and *SPOP* are adjacent to each other on chromosome 17q21.33, and the observation that both are overexpressed in ER+ tumors suggests that these two genes may be co-amplified in most ER+ tumors, as is the case in ER+ cell lines. *NXPH3* encodes neurexophilin-3, which is overexpressed in ER+ tumors (p = 0.0006). Neurexophilin-3, a postulated alpha-neurexin ligand, has never been implicated in cancer, to our knowledge. However, other neurexophilin family members have been implicated in neuroblastoma [22], prostate [23], ovarian [24], and breast [24] [25] cancer. *SPOP*, located within 0.5 Mbp of *NXPH3* on 17q21.33, encodes speckle-type POZ protein. Like NXPH3, SPOP is also significantly overexpressed in ER+ tumors (p = 4.84E-06). *SPOP* has been linked to CUL3-mediated attenuation of signaling downstream of *DAXX* (death-associated protein 6) and hedgehog [26], [27]. The observation that SPOP and the adjacent NXPH3 genes are both amplified in ER+ cell lines and overexpressed in ER+ tumors is consistent with the hypotheses that these two genes may comprise an amplicon that is commonly amplified in ER+ tumors. The fact that both SPOP and NXPH3 regulate degradation of transcription factors that are involved in ER, NOTCH, and DAXX signaling further emphasizes the potential significance of this hypothetical amplicon.

## Discussion

Breast cancer therapy, perhaps to a greater extent than any other field of oncology, is motivated by the concept that genotype predicts therapeutic response. This is not a new concept, but rather has its origins in the observation that expression of the product of the *ESR1* gene (ERα) predicts response to endocrine therapy. More recent developments have linked overexpression of *ERBB2* (HER2) to clinical outcome and therapeutic response. The advent of oligonucleotide-based microarray platforms has facilitated the development of several clinically useful gene expression signatures, which offer the promise of incorporating genomic features other than ER, PR, and HER2 into clinical management of breast cancer patients. One such signature, widely used among researchers but not yet risen to standard of clinical care, has facilitated the stratification of breast tumors into four or five intrinsic subtypes [2]; and there is good evidence to suggest that each of these subtypes exhibits a predictable clinical phenotype [28], [29]. Such findings substantiate the belief, almost an article of faith amongst genomic researchers, that one should be able to develop predictive models that are based upon integration of multiple genomic features. This concept defines the intersection between clinical practice and systems biology.

To date, the integration of multiple genomic features into a cogent mathematical model that predicts cellular phenotype has been frustrated by the fact that the output from analytical platforms that have been used is primarily analog. Consequently, it is often difficult to compare microarray data from different laboratories, or for that matter to compare data from the same laboratory run at different times. How much more difficult, then, to integrate data from hybridization-based analyses of gene copy number, promoter methylation, and mRNA abundance? The advent of massively parallel DNA sequencers holds the promise of overcoming some of these difficulties. The output from sequencers is digital, the signal to noise ratio is high, and dynamic range is great. The computational simplicity of merely counting the number of times a defined sequence tag appears within a particular sample should, in theory, make it possible to develop mathematical models that integrate any number of genomic features, with the expectation that such models will at the very least engender hypothetical predictions about the relationship between the various features that comprise the model.

To build such models, one must first develop a curated, disciplined dataset. The development of such a dataset was our primary objective in the experiments described in this report. We elected to focus on breast cancer because of the historical significance of gene expression and therapeutic response in this disease, as discussed above. We elected to use cell lines, rather than primary tumors, because these cells are readily available to any investigator who wishes to confirm or extend our genomic findings. (Note that all of the experiments described above were done with early passage cells that were purchased from ATCC.) Furthermore, the cell lines that we selected have been studied extensively by many investigators, so there is a strong cellular and molecular background to draw upon for future studies. Critically important to our objectives, we elected to use a single platform for all of our genomic analyses, the Illumina Genome Analyzer, in the belief that a disciplined analytical approach would facilitate our ultimate objective of data integration. Finally, we elected to extract both RNA and DNA from the same cell cultures to minimize potential biological variation that might arise from subtle differences in culture conditions. DNA extracted from these cells was used for DNA-seq and Methyl-seq analysis, whereas polyadenylylated RNA was used for mRNA-seq analysis. Additional features of the mRNA-seq analysis included the generation of long read libraries (longer cDNA than the conventional mRNA-seq protocol) and paired end sequence analysis. Exploitation of the paired end data to quantify splice junctions and to identify novel splicing isoforms and fusion gene products is ongoing at this time.

In this initial analysis we concentrated on three genomic features: mRNA abundance, gene copy number, and CpG island methylation status. The decision to analyze these features as a function of estrogen receptor status was obvious, given the clinical significance of ER as a therapeutic marker. This focus was substantiated by the very robust stratification of the cell lines based on unsupervised clustering of the gene expression data. Several points about the mRNA expression data warrant discussion. Although not a major focus of this report, we compared several different statistical models including ANOVA, negative binomial regression, Poisson regression, and a Bayesian implementation of the modulated t-test that had originally been developed for microarray analysis (LIMMA). As a primary end point, we compared each of these models for the ability to identify genes that were differentially expressed, with the NanoString data considered to be the ‘true’ test of differential expression. A separate manuscript describing this comparison is in preparation, but our analyses indicated that for this dataset, LIMMA was significantly more reliable than any other model. Consequently, we used LIMMA to assess the statistical significance of observed differences in mRNA abundance and CpG island methylation.

The broad dynamic range of mRNA-seq analysis makes it possible to detect transcripts that are present at very low abundance, easily below the range of 1 tag/M. However, our analysis of variance (standard deviation) as a function of expression (total tags) indicates that this level of detection is probably not within the range of reliable quantification. Therefore, we felt it was necessary to exclude from our analysis a subset of 5,320 genes that had average expression levels of <50 total tags (∼1 tag/M) in both the ER+ and ER− groups. We also eliminated genes that had >50 total tags on average, but for which there were <5 total tags in one or more samples within either group. Among these, 580 genes were statistically significant, as assessed by p-value<0.05. Thus we eliminated from our analyses a group of very low abundance genes in which about 10% appeared to be differentially expressed but were of such low abundance that we were not confident of the quantification and meaningful integration with methylation and CNA data. This point warrants additional emphasis: mRNA-seq is capable of detecting transcripts at very low levels, but quantification of such transcripts is problematic and may require more detailed analysis of features such as exon coverage.

We detected 1873 genes that were differentially expressed in ER+ and ER− cell lines, at a modest level of statistical stringency (p<0.05, FDR q<0.2, fold change >1.5). Validation experiments indicate that the mRNA-seq data generally conform to data obtained with two different analytical platforms (qPCR and NanoString). Pathway analysis of these genes revealed statistically significant enrichment of known ER-associated functions. The question then arises of the extent to which these differences in gene expression profile can be linked to copy number aberrations or to differential methylation of CpG islands located in the vicinity of the cognate promoters.

We used segmentation analysis to compare copy number in the tumor cells to that in non-transformed MCF10A cells. The use of MCF10A as a reference standard is debatable, since these are not normal human mammary epithelial cells. However, visual examination of the distribution of CNV-seq sequence counts across all chromosomes in this cell line revealed no notable regions of gene amplification. The calculated chromosomal coverage in our analyses was on average about one tag every 300 bp, corresponding to 100× physical coverage for a gene of 30 kb. As one would expect, we detected many regions that exhibited copy number aberration in each of the tumor cell lines. Most of these were cell line-specific. However, we did identify 479 genes that are differentially expressed in ER+ versus ER− cells and that may be regulated by changes in gene copy number in two or more cell lines of either phenotype. However, we have focused our analysis upon a core of 30 genes that are overexpressed either as a result of amplification of chromosome 17 (in ER+) or deletion of chromosome 8 (in ER−). These features were common to all ER+ or ER− cell lines, suggesting that some of the genes within these loci may be essential to establishment or maintenance of the ER+/ER− phenotypes.

The methylation signatures that we detected in these cells were significantly more robust than the copy number aberrations. We identified some 162 differentially expressed genes that exhibited very highly significant changes in CpG island methylation and for which methylation status correlated inversely with expression. Not surprisingly, the majority of these CpG islands were very close to the 5′ ends of the differentially expressed genes. Our data suggest that a minimum of 10% of the genes that define the ER+/ER− expression profiles are likely to be regulated by promoter methylation. This is almost certainly an underestimate, since our analysis is likely to identify only those genes that exhibit very large changes in methylation status.

Our analyses have identified a subset of 30 genes that are overexpressed in ER+ cells and are either amplified in ER+ or deleted in ER− cells. In addition, we have identified 149 genes that are differentially expressed in ER+ versus ER− cells, differentially methylated on one or more promoter-proximal CpG islands, and exhibit an inverse correlation between CpG island methylation and mRNA abundance. The observation that common mechanisms underlie differential expression implies that some or all of these genes are regulated by global genomic processes that are central to establishment and/or maintenance of the ER+/ER− phenotypes. That hypothesis predicts that this cohort of genes should be enriched in ER+/ER− tumors, and our GSEA analysis of a large microarray dataset from such tumors is consistent with this prediction (p<0.001, q = 0.06). Some 103 of our 179 focus genes were differentially expressed in the tumor dataset. Fourteen of the 30 genes that exhibited CNA in the cell lines were differentially expressed in the tumors; however, only 9/14 were overexpressed in ER+ tumors. Conversely, 84/149 of the differentially methylated genes were coordinately and significantly expressed in both ER+/ER− cell lines and tumors. Included among these were several genes that have been linked to clinical outcome, notably GATA3 (hypermethylated in ER-cell lines and repressed in ER− cell lines and tumors) and LYN (hypermethylated in ER+ cell lines and repressed in ER+ cell lines and tumors).

Our data are correlative in nature, and do not rigorously establish a link between methylation status or copy number aberration in cell lines and expression in tumors. Nevertheless, our data are consistent with the hypothesis that there is a significant subset of differentially expressed genes that are likely to be regulated by such mechanisms and to play important roles in establishment and/or maintenance of the ER+/ER− phenotypes in breast cancer. Copy number aberration may be involved, but our data suggest that only a few genes are likely candidates for ER-specific amplification or deletion. Conversely, CpG island methylation appears to be linked to differential expression of a large cohort of genes that define the ER+ versus ER− tumor phenotype. Some of these genes are known to be functionally significant (*e.g. GATA3* and *LYN*) whereas the functional significance of other genes can only be inferred (*C6orf97*) or is completely unknown (*COPN8*). Overall, however, there is a very strong indication that global methylation patterns are critical to breast tumor phenotypes, including therapeutic response and clinical outcome. Of particular interest are those genes that are hypermethylated in ER+ cells and overexpressed in ER− cells and tumors, since these may include potential therapeutic targets (*e.g.* LYN) that could be exploited to treat ER− (basal/triple negative) tumors.

## Materials and Methods

### Data sharing

All of the sequence data that were analyzed in this report have been deposited in Gene Expression Omnibus (GSE27003).

### Breast cell lines

Eight breast cell lines, 7 from breast cancer and one from non-tumor breast epithelial cells, were obtained from the American Type Culture Collection (ATCC). The characteristics of these cell lines were confirmed by qPCR analysis of ER, PR, and HER2 mRNA. Among the 7 cancer cell lines, 4 are ER+ and 3 are ER−. All cell lines were grown under conditions recommended by ATCC and RNA and DNA were extracted from mid log phase populations of low passage number cultures.

### RNA preparation and sequencing

Total RNA extraction was performed using Exiqon's miRCURY RNA Isolation Kit. Long-read mRNA-seq cDNA libraries were prepared from 1µg of total RNA using a modification of the Illumina mRNA-seq protocol. Briefly, mRNA was resolved using poly-dT oligonucleotides attached to magnetic beads, fragmented using divalent cations under elevated temperatures, and converted to cDNA using random primers. After conversion of the cleaved fragments into cDNA, the cDNA underwent blunt end repair, addition of an ‘A’ base to the 3′ blunt ends, and ligation of adapter molecules which will be used for PCR amplification, bridge amplification, and sequencing. The cDNA library was resolved by gel electrophoresis using conventional Illumina protocols except that we cut from the gel those cDNA fragments in the range of 300–400 bp. The increased fragment length is necessary to accommodate paired end sequence analysis. The gel purified cDNA fragments were amplified by PCR and sequenced using the Illumina Cluster Station and Genome Analyzer. Paired-end sequence analysis (51 cycles/end) was carried out using sequencing primers that correspond to either end of the bridge-amplified cDNA fragments so as to obtain 50 nt of sequence from either end of every cDNA fragment.

### mRNA-seq data analysis

The Illumina standard pipeline version 1.4 was employed for processing of raw images to make base calls and generate sequence reads. Reads were aligned to genome and exon junctions using Illumina's alignment tool Eland_RNA (NCBI36/UCSC hg18). A maximum of two mismatches were allowed for first 32 bases in each alignment, and reads that had more than two mismatches or were mapped to multiple genomic locations (alignment score less than 4) were discarded. The aligned sequence tags were summarized and annotated using Illumina's CASAVA tool (version 1.0) and imported into the Genome Studio software. The read counts for genes, exons, and exon junctions were exported from Genome Studio. A total of 18,517 genes were annotated using RefSeq RNA database and the raw read counts were used for downstream analyses.

The same mRNA library preparation was sequenced from both ends of each cDNA fragment twice (Paired-End sequencing) and the raw read counts from each end were combined for increased coverage. The combined reads for each gene were normalized by the total reads of each individual cell line and then standardized to reads per million (RPM, gene counts/total counts of each cell line ×1 million). For differential gene expression analysis between ER+ and ER− cell lines, we first eliminated genes without any reads across all 7 cell lines. We added 1 to all the genes and samples before converting to RPM so that we could deal with genes with zero count in some of samples to facilitate log2 transformation.

The log2 transformed data were visualized by hierarchical clustering and heat maps for all the genes first and then for a subset of highly varied genes across seven cell lines (standard deviation greater than 75^th^ percentile). The distance matrix was 1-correlation and linkage method was average. Differentially expressed genes between ER+ and ER− cell lines were identified using the linear models for microarray (LIMMA) package in R [30]. This package uses an empirical Bayesian implementation to estimate a standard error and has improved performance when an experiment has a limited number of samples. False discovery rate (FDR) was estimated using q-value [31]. As one of our goals was to explore the underlying causes of differentially expressed genes between ER+ and ER− cell lines from methylation and DNA abnormality perspectives, a generous nominal p-value cut-off of 0.05 was used for significant changed genes to correlate the gene expression with the methylation and DNA copy number changes. For pathway analysis of differentially expressed genes, we applied a more stringent criterion of including only genes with a FDR of q-value less than 0.1. We carried out concordance analysis in which we compared the mRNA-seq data to expression data from the same samples obtained using the NanoString cancer reference gene set data (see below). In this analysis we compared LIMMA, over-dispersed Poisson model, DESeq (negative binomial model), and Student's t-test for identification of differentially expressed genes between ER+ and ER− cell lines. We observed that LIMMA processing of mRNA-seq data gave the highest concordance with NanoString data. Therefore we selected LIMMA for analyzing both mRNA-seq and Methyl-seq data.

### Validation of mRNA-seq with qPCR and NanoString on a set of cancer reference genes

The NanoString nCounter Cancer Reference CodeSet was used to validate mRNA-seq data (http://www.nanostring.com/products/assays/). This codeset contains a 3′ biotinylated capture probe and a 5′reporter probe tagged with a fluorescent barcode, two sequence-specific probes for each of 236 transcripts. Probes were hybridized to 100 ng of total RNA for 19 h at 65°C, after which excess capture and reporter probes were removed and transcript-specific ternary complexes were immobilized on a streptavidin-coated cartridge. All solution manipulations were carried out using the NanoString preparation station robotic fluids handling platform. Data collection was carried out with the nCounter Digital Analyzer to count individual fluorescent barcodes and quantify target RNA molecules present in each sample. Normalization was carried out based on a standard curve constructed using spike in exogenous control samples. Background hybridization signal was determined using spike in negative controls, and all mRNAs had fewer than mean background+2 standard deviations were considered to be below the limits of detection.

The raw code count data from the nCounter Analysis System were first normalized and background corrected. Specifically, a normalization factor was calculated based on the relative number of positive control counts in each sample. Genes with counts less than the average of embedded negative controls (background noise) in that sample were first set to its background. The gene count for each gene was subtracted from this background so that each sample had same footing where zero numbers represent undetectable noise. When comparing the data to mRNA-seq data and detecting differentially expressed genes, we log2 transformed the data after each data point was increased by adding 1 to deal with zeros. Correlation coefficients between the mRNA-seq and NanoString data were determined using Pearson product-moment correlation coefficient. Student's *t*-test was applied to the NanoString data to identify differentially expressed genes between the ER+ and ER− cell lines.

### CpG Island methylation by Reduced Representation Bisulfite Deep Sequencing

DNA (2µg) extracted from cell lines was fragmented using endonuclease *MspI*, followed by QIAQuick purification. The end of digested DNA was repaired and an adenine was added to the 3′ end of the DNA fragments according to the Illumina standard end repair and add_A protocol (Illumina, San Diego, CA). Pre-annealed forked Illumina adaptors containing 5′-methyl-cytosine instead of cytosine was ligated to both ends of DNA fragments using standard Illumina adaptor ligation protocol (Illumina). Ligated fragments were then separated by 2% agarose gel. Two size ranges, 150–175 bp and 175–225 bp (includes adaptor length), were selected and cut from the gel. DNA from gel slices was purified using Qiagen Gel extraction kit (Qiagen). The purified DNA was treated with EpiTect Bisulfite kit (Qiagen) with modification. The bisulfite conversion time was extended to approximately 14 hr by adding 3 cycles of denaturation at 95°C for 5 min followed by incubation at 60°C for 180 min. The bisulfite-converted DNA was purified using the EpiTect Bisulfite kit and the protocol for DNA isolated from formaldehyde-fixed, paraffin-embedded tissue samples. The bisulfite-treated DNA was purified a second time with MinElute PCR purification kit (Qiagen) and eluted with 15µl EB buffer. The bisulfite-treated DNA fragments were PCR amplified: 15µl of eluted DNA, 5 pmol of Illumina PE PCR primers 1.0 and 2.0, 62.5 nM of each dNTP, and 2.5 U of Pfu Turbo Cx hotstart DNA polymerase (Stratagene Products, Agilent, La Jolla, CA) in a total 50µl volume. The amplification conditions were as follows: 5 min at 95°C, 30 sec at 98°C then 6× (10 sec at 98°C, 30 sec at 65°C, 30 sec at 72°C) followed by 5 min at 72°C. The PCR reaction was purified by MinElute PCR purification Kit (Qiagen) and final reduced representation bisulfite library was eluted in 15µl EB. The concentration of final library was measured using the Agilent 2100 Bioanalyzer (Palo Alto, CA). The library was sequenced on Illumina GA sequencing instrument according to standard Illumina cluster generation and sequencing protocols.

DNA sequencing (50 nt) was conducted on one end of the DNA fragments. About 62% of all 50 nt sequence tags were uniquely mapped to the human genome in 3 letter space. A multi-fasta file of sequences for both forward and reverse strands, consisting of 50 nucleotides or less if the next *MspI* site is located less than 50 nucleotides apart, adjacent to *MspI* sites was used as a reference for alignment. A converted reference, where every C was replaced by T for forward strand fragments and every G replaced by A for reverse strand fragments was prepared. All reads from the Genome Analyzer (qseq files from Bustard) were converted into three bases (A,G,T), *i.e.*, simply replacing all remaining Cs with Ts. The converted reads (50 nt) were aligned to converted reference by stand_alone Eland_extended module. The repeat-masking option of Eland was used to mask known multiple hits. The positions from these alignments were used to generate reference sequences from the original (4 bases) *MspI* fragments. The original 4 base reads from Genome Analyzer were matched to the corresponding reference sequences (4 bases). Methylated C base was obtained by counting C/C+T ratio. Summarized methylation data on each CpG island were obtained from averaging all CpG sites with coverage> = 10 in a CpG island. These data represent the percentage of methylated CpGs over total number of CpGs in the island (from 0 to 100). CpG islands within 5 kb of the 5′ end of a gene were included for the analyses and comparisons. The overall profile of methylation was examined using unsupervised hierarchical clustering where distance matrix was 1-Pearson correlation and the linkage method was average. The differentially methylated CpG islands were identified using the LIMMA package, as described for analysis of gene expression. A p-value cut-off of 0.05 was applied for significantly methylated CpG islands.

### DNA preparation and sequencing

Genomic DNA was extracted using Qiagen's QIAamp DNA Mini Kit. Genomic DNA libraries were constructed according to the standard Illumina protocol. Briefly, DNA (5µg) was fragmented using the Covaris shearing apparatus. The end of digested DNA was repaired and an adenine was added to the 3′ of the DNA fragments according to the Illumina standard end repair and add_A protocol. After adaptor ligation using standard Illumina adaptor ligation protocol, ligated fragments were separated by 2% agarose gel and DNA fragments of around 400 bp were selected and purified using Qiagen Gel extraction kit. Size selected DNA fragments were then amplified using standard Illumina PCR amplification protocol with 12 PCR cycles. The concentration of the final library was measured using the Agilent 2100 Bioanalyzer (Palo Alto, CA). The library was sequenced on Illumina GA sequencing instrument according to standard Illumina cluster generation and sequencing protocols. Sequencing was carried out to a depth of ≥10 M aligned tags, which corresponds to a theoretical coverage of about one tag every 300 bp, assuming 3E9bp/genome divided by 1E7 tags/sample.

### DNA copy number aberration detection

Genomic 50 bp single end DNA sequencing data were generated to identify copy number aberrations (CNA) in 7 breast cancer cell lines in reference to the non-tumor epithelial cell line (MCF10A). Tumor cell lines were compared to the reference to obtain log2 ratios using BWA [32] paired-end uniquely mapped reads to the genome. We identified the CNAs using the combination of CNV-seq software [33] and Partek Genomics Suite segmentation algorithm. Specifically, for each sample, we mapped the filtered BWA alignment reads to a chromosome and exact base pair locations for input into the CNV-seq software. CNV-seq software uses a sliding window approach to count number of mapped reads in a region for each sample and these counts are used to calculate log2 values. The log2 count values were normalized at individual chromosome level based on the assumption that most parts of the chromosomes have no copy number changes. After normalization we performed median adjustment to the counts obtained from tumor and reference samples so that the median log2 values for tumor and reference were similar. The log2 ratio between a tumor and a reference was defined by the difference between the median log value of tumor counts and the median log value of reference counts for a given segment. To detect copy number changes, we imported the log2 ratio data into Partek Genomic Suite (www.partek.com) and applied a genomic segmentation algorithm with p-value cutoff at 0.0001 for neighboring regions for significantly different means, 10 minimum number of data points for any candidate region, 0.3 signal to noise difference as minimum magnitude of change, and p-value threshold 0.0001 for one-sided t-test for a changed region (below and above thresholds −1 and 0.59, which is equivalent to log2(1/2) = one copy deletion and log2(3/2) = one copy amplification, respectively). We merged the adjacent CNAs and also obtained overlapped CNAs found in two or more samples using R package CNTools. Genes that reside in the identified CNA regions were retrieved using SQL queries according to their genomic locations. The log2 ratio of that region was used for gene copy number.

### Correlation of mRNA-seq data with methylation and copy number aberration

Genes in the final analysis were merged with methylation data and identified copy number aberration regions according to genomic locations. A scatter plot was created for the genes that were differentially regulated and for which surrounding CpG islands were also differentially methylated. A correlation coefficient between the log2 fold change of gene expression and mean difference of methylation for these genes was calculated using Pearson product-moment correlation coefficient, which ranges from −1 to 1 where −1 and 1 represent perfect negative and positive correlation and 0 for no correlation. A two-sided t-test was conducted to compare the correlation coefficient with 0 and the 95% confident intervals were estimated for the correlation coefficient. The distance of these CpG islands from genes was also plotted against the log2 fold change of differentially expressed gene. In correlating gene expression with DNA copy number aberrations, average log2 ratio for a segment from each group (ER+ or ER−) was used to determine the mean copy number difference between ER+ and ER− groups.

### Pathway analysis for differentially expressed genes

We conducted pathway analysis for the genes with FDR less than 0.1 using the genes kept in the final analysis as a reference list in MetaCore (GeneGo Inc). Both canonical pathways (GeneGo Maps) and GeneGo process networks were evaluated. In both analyses, the uploaded focus gene list was compared to the manually curated and pre-built pathways or biological process networks using hypergeometric test to get an enrichment p-value for each pathway or network. The p-value indicates the possibility of a set of genes that is mapped to a pathway or network by chance.

### Validation of the genes regulated by methylation or affected by CNA in a public dataset

Our integrated analyses identified a set of 179 genes that were regulated by methylation (149 genes) or affected by CNA (30 genes) in cell lines. To examine whether these genes were also differentially regulated in tumor samples, we analyzed a cohort of 129 primary breast cancer gene expression profiles generated using the Affymetrix U133plus2 platform and downloaded from Gene Expression Omnibus (GEO accession number: GSE5460) [8]. The data were log2 transformed and differentially expressed genes were identified using the t-test. To address the question whether the set of 179 genes together and the two sets of 149 and 30 genes separately were significantly enriched in this set of tumor samples, we conducted a gene set enrichment analysis (GSEA) as described by Subramanian [34]. We evaluated these user-defined gene sets along with 1,425 well-curated public gene sets (filtered out 470 gene sets that had fewer than 15 or more than 500 genes). The phenotypic class was ER+ or ER-status of the tumors, and the genes that were overexpressed in ER+ or ER− tumors were evaluated separately. From this analysis, a normalized enrichment score (NES), nominal p-value from 1000 permutations, and FDR q-value adjusting for a gene set size and correlations between gene sets and expression datasets were obtained.

All the analyses other than specifically noted were conducted in R: A language and environment for statistical computing (http://www.r-project.org).

## Supporting Information

Table S1 Differential analysis results for mRNA-seq data. It contains 1,873 genes passing our filtering criteria with log2 fold change, p value, and false discovery rate q value.
(XLSX)

Table S2 Pathway analysis results for 451 differentially expressed genes. Only significantly enriched pathways or networks are listed. Those with bold face have estrogen involvement. P value was from hypergeometric test. The ratio represents the number of differentially expressed genes over the total number of genes in the pathway or network.
(DOCX)

Table S3 Differentially methylated CpG islands and their associated genes (within 5 kb of transcript start). Both methylation and gene expression data are included. Note that there are 444 unique CpG islands differentially methylated, with 469 unique associated genes in the table. Some CpG islands (36) have more than one gene within 5 kb window. Some genes (10) have more than one CpG islands within 5 kb of its transcript start.
(XLSX)

Table S4 149 genes (with highlight) whose gene expression was inversely correlated with CpG island methylation. Both gene expression and methylation were differentially regulated between ER+ and ER− cell lines.
(XLSX)

Table S5 Detected CNA segments in 7 breast cancer cell lines (reference to MCF10A normal cell line).
(XLSX)

Table S6 Gene expression and CNA data for 30 genes that were differentially expressed between ER+ and ER− cell lines and their copy numbers were consistently changed in ER+ or ER− cell lines (amplified in four ER+ cell lines or deleted in three ER− cell lines).
(XLSX)
