# Supplementary material for: Integrated Analysis of Gene Expression, CpG Island Methylation, and Gene Copy Number in Breast Cancer Cells by Deep Sequencing
Source: PLoS One. 2011 Feb 25;6(2):e17490. doi: 10.1371/journal.pone.0017490 (PMC3045451; doi:10.1371/journal.pone.0017490)
Supplement: Table S2 — Pathway analysis results for 451 differentially expressed genes. Only significantly enriched pathways or networks are listed. Those with bold face have estrogen involvement. P value was from hypergeometric test. The ratio represents the number of differentially expressed genes over the total number of genes in the pathway or network. (DOCX) [file pone.0017490.s002.docx]

### Table S2: Significantly enriched pathways and process networks for differentially expressed genes between ER+ and ER- cell lines

| **Order** | **Canonical Pathway** | **pValue** | **Ratio** | **Biological process network** | **pValue** | **Ratio** |
| --- | --- | --- | --- | --- | --- | --- |
| 1 | Cell adhesion_ECM remodeling | 0.000471 | 7/30 | **Signal transduction_ESR1-membrane pathway** | 0.000183 | 10/55 |
| 2 | Immune response_IL-15 signaling | 0.00094 | 8/43 | Inflammation_Jak-STAT Pathway | 0.001198 | 11/81 |
| 3 | **Cytoskeleton remodeling_ESR1 action on cytoskeleton remodeling and cell migration** | 0.001413 | 4/11 | Chemotaxis | 0.001949 | 8/50 |
| 4 | Cytoskeleton remodeling_Thyroliberin in cytoskeleton remodeling | 0.002833 | 4/13 | Cell adhesion_Glycoconjugates | 0.002908 | 9/65 |
| 5 | Regulation of lipid metabolism_RXR-dependent regulation of lipid metabolism via PPAR, RAR and VDR | 0.002833 | 4/13 | Apoptosis_Anti-Apoptosis mediated by external signals via PI3K/AKT | 0.005114 | 11/97 |
| 6 | CoA biosynthesis | 0.005009 | 4/15 | **Apoptosis_Anti-Apoptosis mediated by external signals by Estrogen** | 0.006309 | 7/48 |
| 7 | Neurophysiological process_Dopamine D2 receptor signaling in CNS | 0.005438 | 3/8 | **Signal transduction_WNT signaling** | 0.007366 | 12/116 |
| 8 | Neurophysiological process_ACM regulation of nerve impulse | 0.006427 | 4/16 | Cardiac development_BMP_TGF_beta_signaling | 0.01067 | 9/79 |
| 9 | Immune response_MIF-mediated glucocorticoid regulation | 0.01437 | 3/11 | Inflammation_Interferon signaling | 0.01102 | 8/66 |
| 10 | Neurophysiological process_Thyroliberin in cell hyperpolarization and excitability | 0.02317 | 3/13 | Blood coagulation | 0.01242 | 6/42 |
| 11 | Development_Thrombospondin-1 signaling | 0.02317 | 3/13 | Cell adhesion_Platelet-endothelium-leucocyte interactions | 0.01562 | 9/84 |
| 12 | Development_ACM2 and ACM4 activation of ERK | 0.02394 | 4/23 | Apoptosis_Anti-Apoptosis mediated by external signals via MAPK and JAK/STAT | 0.01744 | 10/100 |
| 13 | Immune response _Immunological synapse formation | 0.02766 | 4/24 | Proteolysis_Proteolysis in cell cycle and apoptosis | 0.01981 | 10/102 |
| 14 | Mitochondrial unsaturated fatty acid beta-oxidation | 0.02845 | 3/14 | **Signal transduction_ESR1-nuclear pathway** | 0.021 | 14/165 |
| 15 | Cell adhesion_Cell-matrix glycoconjugates | 0.02845 | 3/14 | Neurophysiological process_GABAergic neurotransmission | 0.02306 | 6/48 |
| 16 | Arachidonic acid production | 0.02845 | 3/14 | Reproduction_Feeding and Neurohormone signaling | 0.02618 | 11/122 |
| 17 | Development_ERBB-family signaling | 0.03171 | 4/25 | Development_Neurogenesis:Synaptogenesis | 0.02994 | 10/109 |
| 18 | **Transcription_Ligand-dependent activation of the ESR1/SP pathway** | 0.03171 | 4/25 | Apoptosis_Death Domain receptors & caspases in apoptosis | 0.0303 | 9/94 |
| 19 | Vitamin E (alfa-tocopherol) metabolism | 0.03431 | 3/15 | Development_Ossification and bone remodeling | 0.03412 | 8/81 |
| 20 | Mitochondrial long chain fatty acid beta-oxidation | 0.03431 | 3/15 | Cell adhesion_Cell-matrix interactions | 0.03614 | 9/97 |
| 21 | Chemotaxis_Inhibitory action of lipoxins on IL-8- and Leukotriene B4-induced neutrophil migration | 0.04075 | 3/16 | Inflammation_Innate inflammatory response | 0.03635 | 8/82 |
| 22 | Neurophysiological process_ACM1 and ACM2 in neuronal membrane polarization | 0.04075 | 3/16 | Apoptosis_Apoptosis stimulation by external signals | 0.04271 | 9/100 |
| 23 | Transport_Alpha-2 adrenergic receptor regulation of ion channels | 0.04075 | 3/16 | Transcription_Nuclear receptors transcriptional regulation | 0.04317 | 11/132 |
| 24 | Inhibitory action of Lipoxins on neutrophil migration | 0.04075 | 3/16 | Transport_Synaptic vesicle exocytosis | 0.04626 | 8/86 |
| 25 | Development_Thyroliberin signaling | 0.0408 | 4/27 | Cardiac development_FGF_ErbB signaling | 0.04899 | 8/87 |
| 26 | Apoptosis and survival_Lymphotoxin-beta receptor signaling | 0.04584 | 4/28 |  |  |  |
| 27 | Development_Role of CDK5 in neuronal development | 0.04775 | 3/17 |  |  |  |
